# ABO/Rhesus blood group systems and malaria prevalence among students of the University of Dschang, Cameroon

**DOI:** 10.5281/zenodo.10797079

**Published:** 2016-04-15

**Authors:** Roland Bamou, Silas L. Sevidzem

**Affiliations:** 1 Vector-Borne Infectious Disease Unit, Laboratory of Applied Biology and Ecology (VBID-LABEA), Department of Animal Biology, Faculty of Science, University of Dschang, P.O. Box 067, Dschang, Cameroon.

## Abstract

**Background:**

A study was carried out on students of the University of Dschang, Cameroon, to examine the relationship between ABO blood group, rhesus factor and prevalence of *Plasmodium falciparum* infection.

**Materials and methods:**

Blood group and rhesus factor were typed by agglutination using antisera while malaria infection was determined using Rapid Diagnostic Test CareStart malaria HRP2 *pf.* Out of 620 students 582 were screened for ABO blood group and Rhesus factor, and 276 were tested for *P. falciparum* infection.

**Results:**

Faculty of Science (FS) members and male students were highly represented, with 356 (61.2% ) and 303 (52.1%) participants, respectively. Blood group O was most common (48.8%), followed by blood group A (25.8%), B (23.0%) and AB (2.4%). Total percentage of rhesus positive was 92.4%, and its distribution varied across ABO blood groups. Of the 276 students examined for malaria infection, 27 were found positive (9.8%). Except for blood group AB individuals, of which none were infected, malaria infection did not vary among blood groups.

**Conclusion:**

Rhesus factor and blood group did not impact on malaria infection in the hypo-endemic highland area of Dschang, Cameroon.

## 1 Introduction

About 694 million people in Africa are estimated to be at risk of malaria, which represents 21% of the global population at risk. According to the September 2015 WHO weekly epidemiological record, there were about 214 million cases of malaria and 438,000 deaths in that year [1]. Cameroon is one of the Central African countries suffering intensely from this disease burden, and Dschang is not exempted. Dschang University is located in the heart of Dschang town, which is hypo-endemic for *Plasmodium falciparum* malaria. Resistance to malaria is characterised by the development of an immune response by the host and also depends on innate features possessing protective value against infections. Such features include sickle cell trait (HbAS), sickle cell disease (HbSS) [2-4], ABO blood group type [5,6] and the level of G-6-P-dehydrogenase activity [7]. There are ~30 human blood group types currently known [8], but the ABO and rhesus blood systems are clinically the most important. The ABO blood groups consist of A, B and H carbohydrate antigens, which can regulate protein activities during infection and against these antigens [9]. The rhesus system blood groups consist of rhesus-positive and rhesus-negative on the basis of the presence or absence of rhesus antigens on the red blood cell surface. A study on the association of ABO/ Rhesus systems with prevalence [10] revealed that in different ABO blood groups, the rhesus-positive and rhesus-negative distribution varies among the four ABO blood groups. Although many studies have been carried out at hospital or homestead level, few have focused on this topic in a University setting. A trial out in a Nigerian University (Igbinedion University Okada, Nigeria) confirmed that malaria parasitaemia occurred more frequently in ABO blood group O individuals [11] and that a higher malaria parasitaemia occurred in female than in male students. Here, we focus on genetic and pathogenic mechanisms described by Rowe *et al.* [12] and Fry *et al.* [13] who reported that non-blood group O cases were at increased risk of severe malaria. Recent studies in which pregnant women and children were tested revealed that blood group O confers more resistance to malaria infection than other blood group types [4,14], whereas blood group A has been shown to be detrimental [15]. Although an HIV screening exercise [16] is common at the University of Dschang, little attention has been paid to malaria. Our study aimed to understand the relationship between ABO/rhesus blood systems and malaria prevalence among University students of the University of Dschang attending the Faculty of Science Open Door Day (FSODD). We also considered demographic data, such as sex and faculty of study, as baseline data for sensitization and prediction of control measures of the disease in this setting.

## 2 Materials and methods

### 2.1 Study area and population

This study was conducted on the principal campus (Campus A) of the University of Dschang during the FSODD in June 2015. The University of Dschang (5o27’N, 10o04E) is elevated at an altitude of 1400 m on the Bamileke plateau. The topography is characterised by the juxtaposition of small hills furrowed by little streams flowing towards swampy lakes, with an average temperature of 20.5±6oC. In addition, Dschang is partially an agricultural area, harbouring many swampy areas and valleys with pools of water that favour the development of mosquitoes during the rainy season. Crops and wells in this campus and around houses pose good breeding grounds and resting places for anophelines. During this open-door day, students of the University of Dschang visiting the Animal Biology Stand were individually registered and screened. For each potential participant, explanations about the study were made and oral informed consent was obtained from each participant.

### 2.2 Ethical approval

This study obtained an approval from the Faculty and Departmental Staff Committee, and malaria-positive cases were treated with anti-malaria drugs, as recommended by WHO.

### 2.3 Determination of ABO blood group, Rhesus factor and prevalence of malaria infection

Capillary blood was collected by finger-pricking, using 70% isopropanol and sterile, disposable lancets and cotton. ABO and Rhesus blood grouping were performed using the slide method. A drop of blood from each student was placed on a clean slide in four concentric zones. A drop of each of the antisera, anti-A, anti-B, anti-AB and anti-D, was added and mixed with each blood sample with the aid of a sterile stick. Blood groups were determined on the basis of agglutination. CareStart malaria HRP2 *pf* was performed using the same blood sample of participants following the manufacturer’s instructions.

### 2.4 Statistical analysis

Data were entered in Microsoft Excel 2007, and XLSTAT was used for statistical analysis. Descriptive statistics were performed and Chi-square was used to compare Rhesus factor and prevalence of malaria infection within ABO blood groups. A P-value ≤ 0.05 was regarded as statistically significant.

## 3 Results

More than 620 students visited the Animal Biology departmental stand, of which 582 accepted to participate in the study. The distribution of participants according to gender and faculty was recorded (Table 1). From this table, students of the Faculty of Science (FS), especially males, were highly represented with 356 (61.16%) and 303 (52.06%) participants, respectively. The majority of participants (92.44%) tested positive for Rhesus factor.

**Table 1.** Distribution of participants across university faculties and gender. FS; Faculty of Science; FEMS; Faculty of Economics and Management Sciences; FLPS; Faculty of Law and Political Sciences; FLHS; Faculty of Letters and Human Sciences; FAAS; Faculty of Agronomy and Agricultural Sciences. Numbers in bracket indicate prevalence in each blood group.

	Faculty (%)						
Gender	FS	FEMS	FLPS	FLHS	FAAS	Unknown	Total
Male	203 (35.36)	31 (5.57)	15 (2.6)	30 (5.23)	14 (2.44)	10 (1.74)	303 (52.79)
Female	153 (26.65)	56 (9.76)	17 (2.96)	31 (5.40)	4 (0.69)	10 (1.74)	271 (47.21)
Total	356 (62.02)	87 (15.16)	32 (5.6)	61 (10.63)	18 (3.14)	20 (3.48)	574 (100)

Considering the distribution of blood group and Rhesus factor (Table 2), there was no significant relationship between the distribution of blood group between male and female students. Distribution of Rhesus-positive and - negative cases varied with ABO blood groups (χ^2^=12.21; *df*= 3; *P*=0.007). *P. falciparum* prevalence was 9.78%, and more students with blood group O were infected (15 cases, corresponding to 10.63%). There was no relationship between ABO blood group and *P. falciparum* prevalence (χ^2^=1.093; *df*=3; *P*=0.78) (Table 3), and all students tested positive for *P. falciparum* infection were asymptomatic.

**Table 2.** Frequency of blood gr oup types.

	Blood group (%)				
Rhesus	A	B	AB	O	Total
Positive	140 (24.06)	125 (21.48)	13 (2.23)	260 (44.67)	538 (92.44)
Negative	10 (1.72)	9 (1.55)	1 (0.2)	24 (4.12)	44 (7.56)
Total	150 (25.77)	134 (23.02)	14 (2.41)	284 (48.79)	582 (100)

**Table 3.** Malaria prevalence among blood groups.

	Blood group				
	A	B	AB	O	Total
Number tested	60	69	6	141	276
Malaria positive	6	6	0	15	27
% within population size	2.17	2.17	0	5.43	9.78
% within blood group type	10.0	8.69	0	10.63	9.78

## 4 Discussion

In this study mostly FS students (a faculty with a majority of males) participated, which was probably due to the fact that FS students were better informed about this event. The frequency of ABO blood group distribution varied in a similar way to other countries, with blood group O most common, followed by A, B and then AB [4,11,17,18]. In other reports, the ABO blood group pattern differed even if blood group O was most common [19-21]. According to Cserti and Dzik [22], a distribution pattern of blood group O followed by A is characteristic for African endemic for malaria; the distribution of blood groups is geographically and ethnically dependent.

In addition to ABO blood groups, Rhesus-positivity was dominant with 92.44%. This is similar to previous findings [11,17,23]. The prevalence of 7.56% Rhesus-negative participants is not weak and should not be ignored, given its implications in abortion and haemolytic disease of the newborn [24].

Twenty-seven positive cases were recorded out of 276 tested students, corresponding to a prevalence of 9.77%. This low prevalence of malaria infection among the students might be due to different control measures implemented by the government, such as indoor residual spraying, and window/door screening. However, this could also be due to the geographical position or situation of the locality. Dschang is hypo-endemic for malaria and is located at 1400 m elevation, with low average temperatures that are unfavourable for mosquito development. On the other hand, malaria prevalence was higher than that of Ngala Fambe [23] in Dschang. This is probably due to the fact that malaria infections are seasonal in this area, and most frequent in the period between June and August [23]. There was no significant relationship between the prevalence of malaria and ABO blood groups. According to Wolofsky *et al.* [25], *P. falciparum* sporozoites invade and mature irrespective of the different ABO blood groups [20]. However, other studies have reported variable associations between malaria infection and ABO blood groups [12,13,19,21,26]. This variation could be due to geographical location and/or malaria endemicity.

Our results should be interpreted with care because the sample size was small compared to other similar studies; the reason for this was that not all students were aware of the FSODD and its free screening activities, the few students that were aware and showed up were significant for the visit days. Some other known malaria-protective host polymorphisms such as sickle cell trait (HbAS) have been considered by some authors as most protective against severe malaria [4], but the present study only considered the relationship of malaria prevalence with respect to ABO/Rhesus blood group systems (the clinically most important blood group systems) among the University of Dschang students. All screened students were asymptomatic, which could be another reason why no relationship was found between ABO blood groups and malaria prevalence.

## 5 Conclusion

In 582 students of five faculties blood group O was most common, followed by A, B and AB; Rhesus-positive samples were more prevalent, and a malaria prevalence of 9.77% was recorded. No relationship was found between malaria infection prevalence and ABO blood groups.
